# Resistance-breaking strains of tomato spotted wilt virus hamper photosynthesis and protein synthesis pathways in a virus accumulation-dependent manner in *Sw5*-carrying tomatoes

**DOI:** 10.1038/s41598-025-88028-x

**Published:** 2025-01-29

**Authors:** Maria Isabella Prigigallo, Ugo Picciotti, Giovanni Bubici

**Affiliations:** Istituto per la Protezione Sostenibile delle Piante, Consiglio Nazionale delle Ricerche, via Amendola 165/A, 70126 Bari, Italy

**Keywords:** Tomato spotted wilt Orthotospovirus, *Tospovirus*, Transcriptomics, Viral disease, Biotic, Plant sciences, Plant stress responses

## Abstract

Tomato spotted wilt virus (TSWV; *Orthotospovirus tomatomaculae*) is one of the major horticultural threats due to its worldwide distribution and broad host range. In Italy, TSWV is widely spread in tomato (*Solanum lycopersicum*) crops and causes severe yield losses. In the last decades, several tomato varieties carrying the *Sw-5b* gene for resistance to TSWV have been released. We investigated the interaction between *Sw-5b*-carrying tomatoes and *Sw5*-Resistance-Breaking (SRB) TSWV to elucidate the molecular mechanisms underlying resistance breakage. Transcriptome sequencing (RNA-Seq) was used to analyze 18 tomato leaf samples collected from a field crop naturally infected by SRB TSWV in Italy. An increase in virus accumulation level in leaf tissues (titer) resulted in a higher number of differentially expressed genes (DEGs), ranging from 33 to 44% of the whole transcriptome, when the samples with the lowest and the highest virus titer were compared to the asymptomatic sample, respectively. Photosynthesis and protein biosynthesis were the main down-regulated biological processes, while enzyme families such as oxidoreductases and transferases, genes related to the response to biotic stimuli, solute transport, and vesicle trafficking were overall up-regulated. Remarkably, the expression of around 45% of genes (ca. 14000) of the whole transcriptome was significantly (*P* < 0.05) correlated (positively or negatively) to the virus titer, and in 6% of cases (about 2000 genes) the correlation was high (i.e., absolute value of R^2^ > 0.85). This phenomenon was also verified on 15 genes by a quantitative reverse transcription PCR assay on a greenhouse experiment with a different *Sw-5b*-tomato variety artificially inoculated with another SRB TSWV strain. In conclusion, the tomato transcriptome was considerably rearranged upon TSWV infection, with deregulation of photosynthesis, protein biosynthesis, and induction of defense pathways. Finally, this research demonstrated that the magnitude of transcriptional changes was proportional to the virus accumulation level in the leaves.

**Supplementary Information:**

The online version contains supplementary material available at 10.1038/s41598-025-88028-x.

## Introduction

 Tomato spotted wilt virus (TSWV; *Orthotospovirus tomatomaculae*, family *Tospoviridae*; International Committee on Taxonomy of Viruses (ICTV)^1^) is one of the major horticultural threats due to its worldwide distribution and broad host range^2^. In Italy, TSWV is significantly affecting tomato crops (*Solanum lycopersicum*) in the open field because of its spread and economic impact on yield^3^. This virus is efficiently transmitted by several species of thrips including *Frankliniella occidentalis*(Pergande) (Thysanoptera: Thripidae)^4^. The wide host range, including pepper, tomato, eggplant, broad bean, lettuce, etc.^5^, and the difficulty in deploying effective control measures against its vectors still represents a great challenge for the control of the TSWV-induced disease.

During the past three decades, natural resistance sources against TSWV have been identified and introduced into new commercial tomato varieties^6–8^. The *Sw-5b*, isolated from *Solanum peruvianum* Mill., has been the most widely used gene in tomato breeding programs because of the durability and stability of the resistance conferred against TSWV and other *Orthotospovirus *species^9–13^. Nevertheless, the large use of *Sw-5b*-carrying varieties resulted in the emergence of *Sw-5b*-Resitance-Breaking (SRB) TSWV strains worldwide^14–17^. Moreover, the occurrence of mixed viral infections in the field has been shown to interfere with the resistance mechanisms regardless of the genetic background of the tomato cultivar^18^. This evidence has enhanced the need for alternative management strategies for TSWV disease^15,19^, such as the combined use of genetic resistance and grafting^20^.

RNA-Seq represents a well-established tool to investigate host-pathogen interactions and identify host genes involved in the response to an infection. In the last decades, several studies have elucidated a nearly complete picture of the transcriptomic responses of host plants to different viral infections^21–27^. However, to date, few studies have characterized the transcriptional responses of tomatoes to TSWV infection^24,28–30^. A microarray assay showed how TSWV differentially affected the gene expression in the shoots and roots of tomatoes^25^. Some years later, Padmanabhan, et al.^29^ investigated the molecular basis of TSWV resistance in tomato lines nearly isogenic for the *Sw-7 *gene, another resistance locus in tomatoes. The modulation of several metabolic pathways by viral infection usually causes host-specific symptoms leading to reduced agronomic performance and biomass of the crop. In particular, it has been widely reported that virus-challenged plants show a sharp decline in photosynthesis capacity with dramatic changes in chloroplast components and architecture^31^.

In the present work, we evaluated the gene expression profile of leaves collected from a field crop of a *Sw-5b*-carrying tomato cultivar in the Apulia region, Italy, in response to a natural infection of an SRB-TSWV strain. Our research provides a better understanding of TSWV-tomato interaction and elucidates the molecular mechanisms behind the resistance breakage in *Sw-5b*-tomato genotypes by SRB-TSWV. Moreover, the transcriptomic analysis revealed that the plant response was strongly correlated to TSWV accumulation level.

## Results

### Complete genome of TSWV FGtomSRB strain

The complete genome of the TSWV FGtomSRB strain was obtained from RNA-Seq data. The mapping of sequencing reads to this genome revealed that 100% of sequencing reads contained the C118Y mutation (TAT codon) in the *NSm *gene, responsible for the SRB trait^5^ and thus indicating that only SRB strain(s) and no wild-type strain(s) occurred in the sampled leaves. Other polymorphisms such as C118F, D122G, T120N, other known mutations^32–35^, or new unknown ones were not observed in the *NSm* gene.

No other viruses were detected in the samples using a bioinformatic pipeline based on contig assembling and searching against the BLAST database.

### The magnitude of transcriptome changes increases with the TSWV accumulation level

**Fig. 1:**
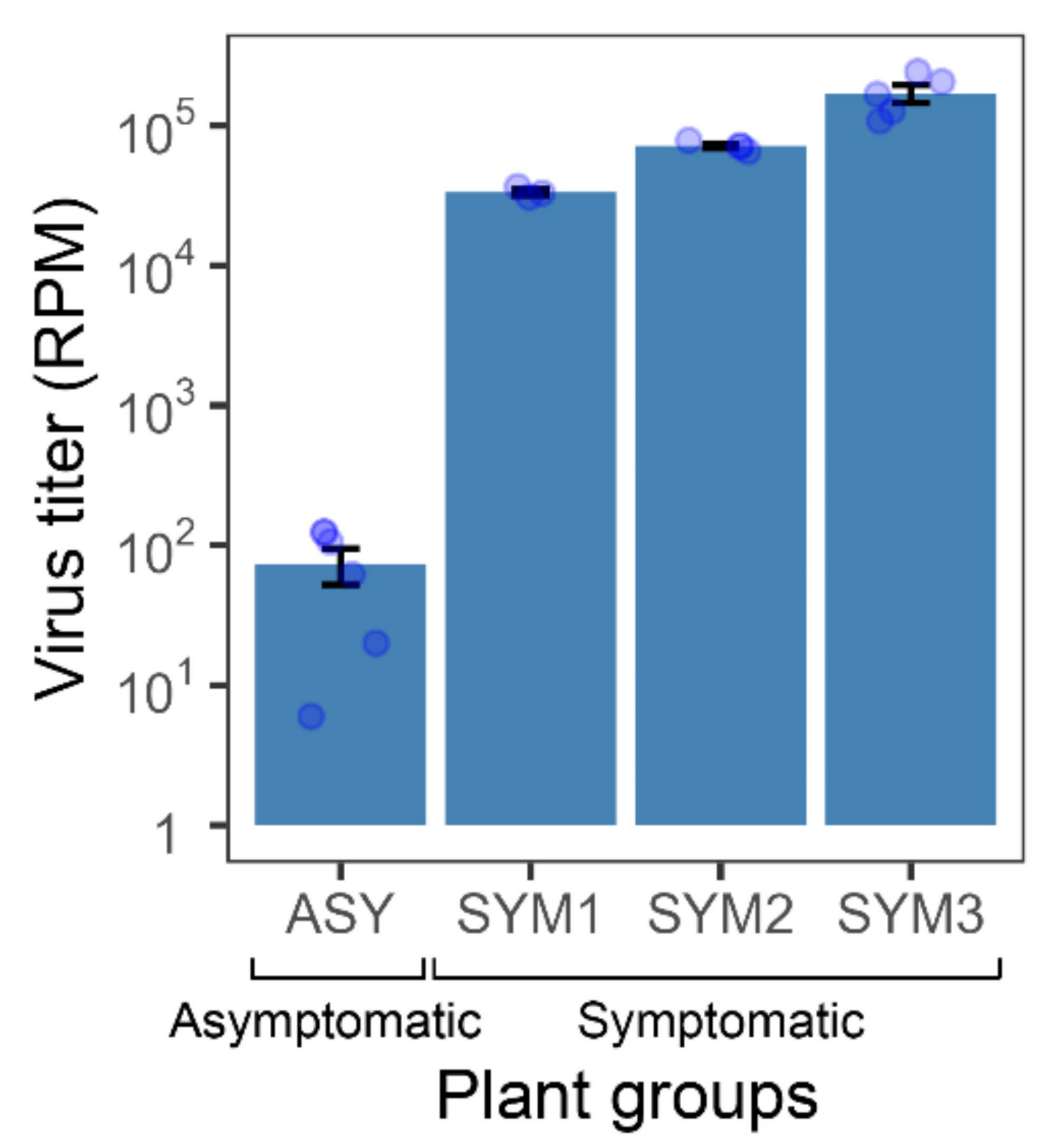
Accumulation of tomato spotted wilt virus (TSWV) in leaves of tomato plants sampled from the field. Virus titer is expressed as reads per million (RPM) mapping to the TSWV genome. ASY plant group included asymptomatic leaf samples with very low virus titer. SYM1, SYM2, and SYM3 groups were symptomatic and differed in virus titer. Further details are shown in Figure S1.

RNA-seq analysis was conducted on 18 tomato leaves collected from symptomatic and asymptomatic plants. The analysis of virus accumulation in the leaf samples, or virus titer (i.e., identification of reads mapping to viral genome normalized by sequencing depth) revealed the presence of TSWV reads in the 12 symptomatic samples ranging from 3.1 × 10^4^ to 2.4 × 10^5^ reads per million (RPM; Figure S1). Based on this result, the symptomatic samples were clustered into three groups according to the virus titer: SYM1 (three samples), SYM2 (four samples), and SYM3 (five samples), with average virus titers of 3.3 ± 0.2·10^4^, 7.2 ± 0.3·10^4^ and 1.7 ± 0.2·10^5^ RPM, respectively (Fig. 1 and S1). Moreover, the six asymptomatic samples were not free from TSWV reads, though they were detected at very low levels, viz. 6 to 1.2 × 10^2^ RPM. These samples were considered as one group namely ASY, with an average virus titer of 0.7 × 10^2^ RPM. Differential gene expression was performed for SYM1, SYM2, and SYM3 as compared to ASY.

**Fig. 2:**
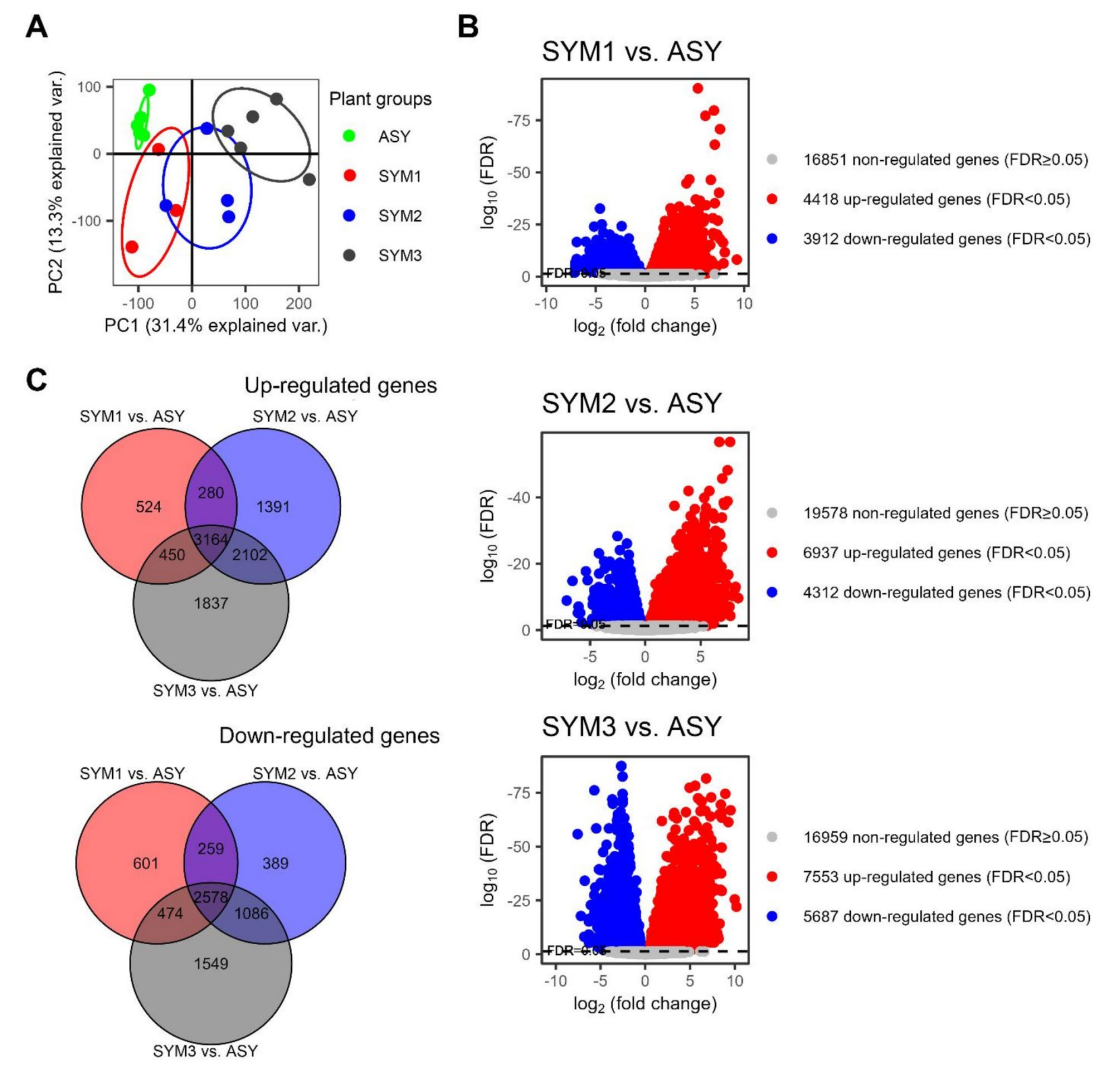
Overview of the RNA-Seq results from the field experiment: principal component analysis (**A**), volcano plots (**B**), and Eulero-Venn diagrams (**C**). Plant groups ASY, SYM1, SYM2, and SYM3 differed for the titer of tomato spotted wilt virus, as depicted in Fig. 1.

Principal component analysis from the RNA-Seq experiment clearly showed the separation of ASY, SYM1, SYM2, and SYM3 groups (Fig. 2A), indicating a remarkable overall difference among these samples. Furthermore, high numbers of differentially expressed genes (DEGs; false discovery rate or FDR < 0.05) were detected in the aforementioned groups. Volcano plots (Fig. 2B) showed that a total of 8330 DEGs were estimated in SYM1, with 4418 up-regulated and 3912 down-regulated. In SYM2, the number of DEGs increased to 11249, with 6937 up-regulated and 4312 down-regulated. SYM3 showed the highest number of DEGs, with 7553 up-regulated and 5687 down-regulated. The Eulero-Venn diagrams (Fig. 2C) also showed that the number of DEGs depended on the virus titer for both up- and down-regulated genes (with the sole exception of the down-regulated genes in SYM2): up-regulated DEGs were 524 in SYM1, 1391 in SYM2, and 1837 in SYM3, and down-regulated were 601, 389, and 1549 SYM1, SYM2, and SYM3, respectively. In addition, 3164 and 2578 DEGs were up- and down-regulated, respectively, in all the comparisons to control (thus placed in the center of the Venn diagram; Fig. 2C).

### TSWV triggers the responses to stimuli while hampering photosynthesis, protein biosynthesis, and gene expression pathways

**Fig. 3:**
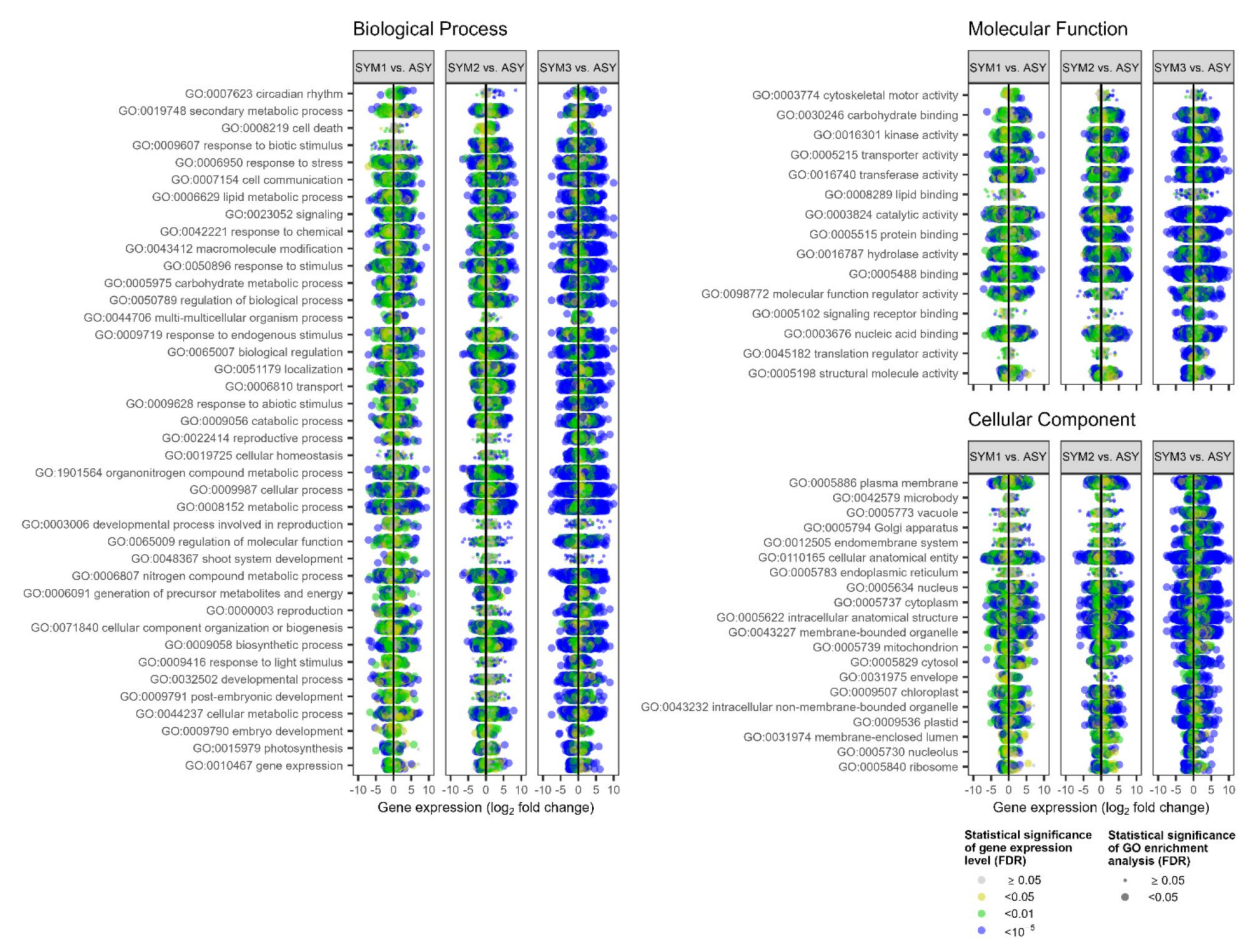
Ontology of genes differentially expressed in tomato plants as revealed by RNA-Seq. Plant groups ASY, SYM1, SYM2, and SYM3 differed for the titer of tomato spotted wilt virus as depicted in Fig. 1.

Transcriptome sequencing revealed that plant response to TSWV infection was significantly related to the virus accumulation level in the leaf tissues. Functional analysis identified 75 gene ontologies (GOs) in biological process (BP), molecular function (MF), and cellular component (CC) groups of GO (Fig. 3). Overall, 40 GOs were over-represented in BP, 15 in MF, and 20 in CC. In almost all GOs, a progressive increase of statistical significance (i.e., lower FDR and larger size of the circles) occurred as the virus titer increased, viz. from SYM1 to SYM3. Nevertheless, the average gene expression level (log_2_ fold change) did not change markedly in the three plant groups.

The cellular anatomical entity GO (GO:0110165; CC) showed the highest number of DEGs in SYM2 and SYM3 (8291 and 6838, respectively). In the BP category, GO:0006950 (response to stress), GO:0009607 (response to biotic stimulus), and GO:0050896 (response to stimulus) displayed the highest number of up-regulated DEGs and showed an increasing number of DEGs as a function of the virus titer. GO:001597 (photosynthesis) and GO:0010467 (gene expression) were the GOs with the highest number of down-regulated DEGs in BP. GO:0045182 (translation regulator activity) and GO:0005198 (structural molecule activity) showed the highest number of DEGs in MF, while GO:0005730 (nucleolus, CC) and GO:0005840 (ribosome, CC) in the CC category.

The Kyoto Encyclopedia of Genes and Genomes (KEGG) analysis confirmed the positive correlation between the number of DEGs and the virus titer. A total of 20 enriched KEGG pathways and 4 KEGG Brite were identified. Membrane transports, xenobiotic biodegradation, cellular community, metabolism of other amino acids, and lipid metabolisms were the pathways with the largest number of up-regulated DEGs (Figure S2). In addition, energy metabolism, replication and repair, and translation exhibited the highest number of down-regulated DEGs. Brite mapping showed enrichment of signaling and cellular processes, metabolism, orthologs, and genetic information processing (Figure S2).

Gene expression analysis associated with MapMan annotations confirmed the repression of photosynthesis, protein biosynthesis, and the increase of down-regulated DEGs in these pathways in all plant groups. In fact, highly significant (*P* < 10^−5^), down-regulated DEGs were more abundant than up-regulated ones (blue circles in Figures S3, S4, and S11). The phosphorylation process, including genes of photosystems, linear electron flow, and ATP synthase complex, was negatively affected by TSWV infection as well as those of the Calvin cycle, especially the RuBisCo ones (Figure S4).

Protein biosynthesis was also hampered, especially at ribosome biogenesis and organelle machinery levels (viz., plastidial and mitochondrial ribosome biogenesis, initiation, elongation, and termination of translation; Figure S5). Moreover, genes devoted to protein modification, including protein folding and peptide maturation, were negatively affected by the virus (Figure S6). On the other hand, a high number of genes related to the phosphorylation process, especially the tyrosine kinase-like (TKL) protein kinase superfamily, were up-regulated (Figure S6). RNA biosynthesis, and hence transcriptional regulation, was also noticeably affected by the viral infection (Figure S7).

TSWV also impacted phytohormone pathways (Figure S8). Several DEGs related to jasmonic acid were activated while genes related to auxins, especially those involved in perception and signal transduction, underwent a strong down-regulation. TSWV induced several abscisic acid-related DEGs and ethylene biosynthetic genes. The expression levels of cysteine-rich peptides- (CRP) and non-CRP-related genes were significantly altered by TSWV (Figure S8).

In the secondary metabolism, the terpenoid pathway, especially carotenoid biosynthesis, exhibited the lowest FDR scores (Figure S9). Finally, most DEGs coding for oxidoreductases (EC 1) and transferases (EC 2) were strongly up-regulated (Figure S10). Several other pathways were also afftected by TSWV, including redox homeostasis, protein translocation, vescicle trafficking, solute transport, etc. (Figures S11-S18).

### Plant response to TSWV infection was significantly related to its accumulation level

**Fig. 4:**
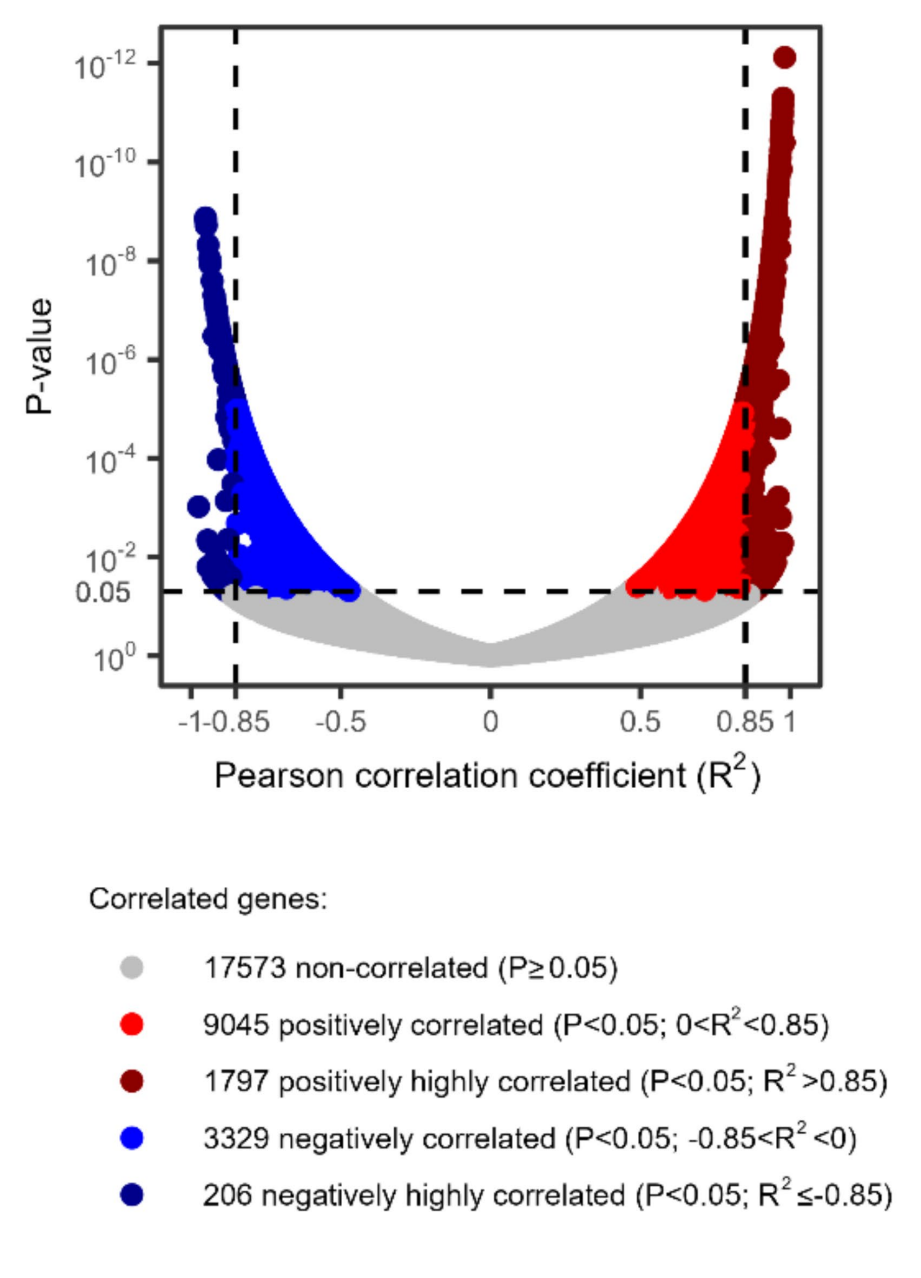
Volcano plot of the correlations between expression of tomato genes and tomato spotted wilt virus accumulation in tomato leaves from the field experiment.

Correlation analysis demonstrated that a large part of the tomato transcriptome changes was strictly related to the TSWV accumulation level in the leaf tissues (Fig. 4). The expression level of 14377 DEGs was significantly (*P* < 0.05) correlated to TSWV titer. In other words, ca. 45% of the whole transcriptome (i.e., 14377 out of 31950 genes) was significantly affected by TSWV infection. The correlation was positive for 10842 genes and negative for 3535 genes. Among these genes, the correlation was high (absolute R^2^ > 0.85) for 1797 and 206 genes positively and negatively correlated, respectively.

### RT-qPCR assay confirmed the gene expression × virus titer correlation observed with the RNA-Seq data

For the RT-qPCR assay, using a tomato variety and an SRB-TSWV strain different from those used in the RNA-Seq experiment, five groups of six seedlings each were inoculated on different days to obtain diverse virus titer on the sampling day (the same for all plant groups). Plants of groups B and C, inoculated on April 28^th^ and May 2^n^d, respectively, showed a fast development of spotted wilt disease as its severity was around 3 (on a 0–4 scale) at 10 dpi, and 4 at 18 dpi (Figure S19A). In the D and E groups, inoculated on May 4^th^ and 5^th^, respectively, the disease progressed more slowly, as its severity was below 0.5 at 10 dpi and 3 at 18 dpi (Figure S19A).

Leaves were sampled on May 10^th^, i.e., 12, 8, 6, and 5 dpi for B to E plant groups. At that time point, TSWV symptoms were observed only in B and C plants (upper panel in Figure S19B). However, disease progression was monitored even after leaf sampling, and TSWV symptoms further developed (lower panel in Figure S19B). As expected, the highest virus titer occurred in B plants and the lowest in E plants, though in E and D groups it showed high variability (Figure S19C). For this reason, 12 of the 30 seedlings were chosen to obtain a regular virus titer gradient across plants ranging from 0 to 3.2 × 10^5^-fold change (Figure S19D).

**Fig. 5:**
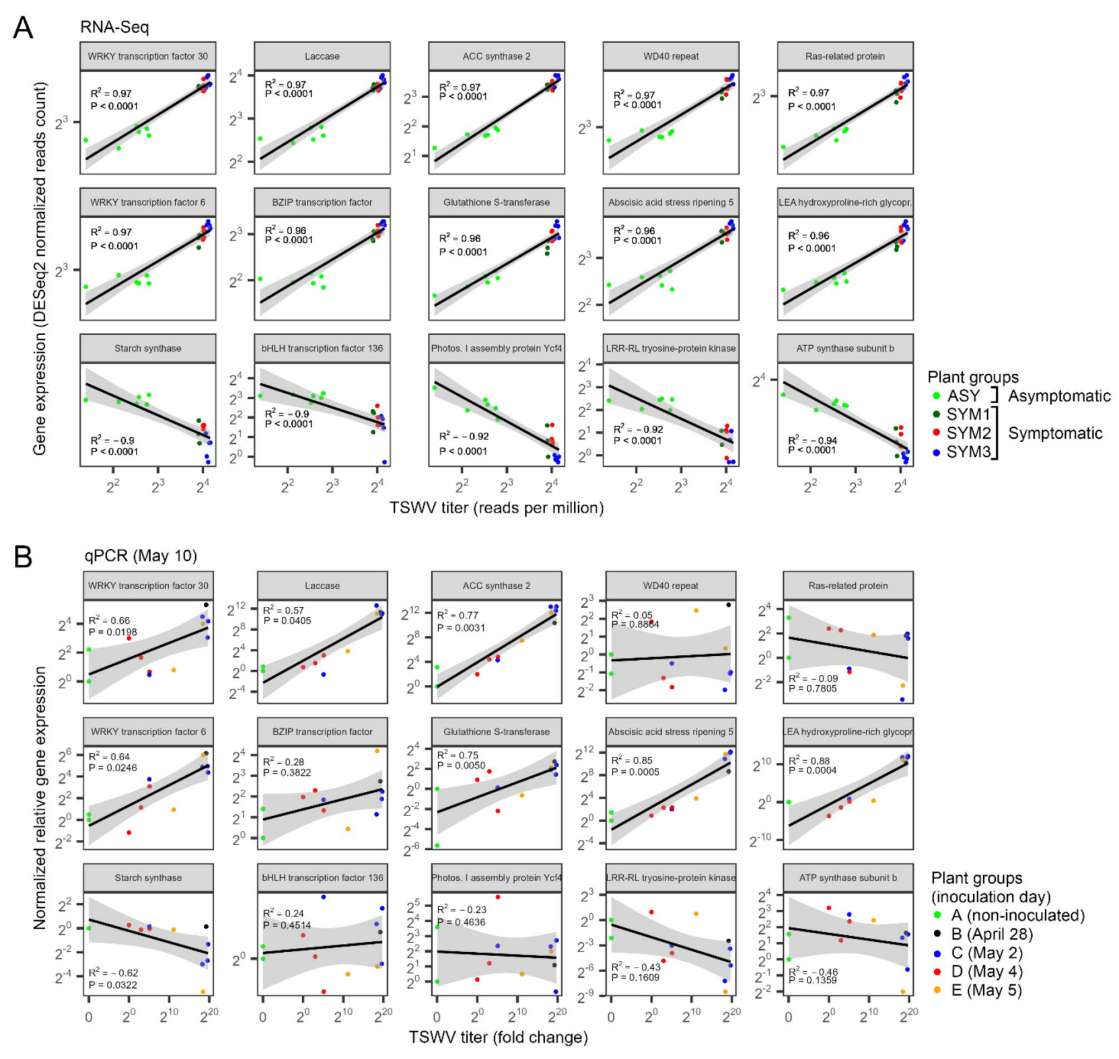
Correlations between expression of tomato genes and tomato spotted wilt virus accumulation in tomato leaves in two experiments: field experiment (**A**), analyzed by RNA-Seq, and greenhouse experiment (**B**), analyzed by RT-qPCR.

Correlation trend (positive or negative) was confirmed for 13 out of 15 genes, and significant (*P*< 0.05) for eight genes, though R^2^ values were overall lower in the RT-qPCR assay. In particular, *WRKY transcription factor 30*, *Laccase*, *1-aminocyclopropane-1-carboxylic acid (ACC) synthase 2*, *WRKY transcription factor 6*, *Glutathione S-transferase*, *Abscisic acid stress ripening 5*, and *Late embryogenesis abundant (LEA) hydroxyproline-rich glycoprotein* were positively and significantly correlated (Fig. 5B). Among the negatively correlated genes, significant correlation occurred only for *Starch synthase*: in the RNA-Seq experiment the correlation of this gene had *P* < 0.0001 and R^2^=−0.9 (Fig. 5A), while in the RT-qPCR assay, it showed *P* = 0.0032 and R^2^=−0.62 (Fig. 5B).

## Discussion

Recently, transcriptome analyses have been reported to identify differentially expressed genes and to study comprehensively the molecular mechanisms underlying plant-virus interactions^21–27^. Here, we investigated the interaction between *Sw-5b*-carrying tomatoes (a TSWV-resistant variety) and SRB TSWV to elucidate transcriptional changes due to the viral infection. This work unveiled the metabolic pathways affected by TSWV infection, proved that the transcriptional response was strictly correlated to virus accumulation in leaf tissues, and identified several genes as good markers of TSWV infection.

Tomato leaves were sampled from a field under natural infection by SRB isolate(s) to compare transcriptomes of infected versus non-infected plants. Nevertheless, RNA-Seq data analysis revealed that asymptomatic leaves were not free from TSWV, and the 12 symptomatic samples showed a large variability of virus titer. The analysis of nucleotide polymorphisms in the *NSm* gene revealed that only SRB TSWV occurred in sampled leaves and the SRB trait was due to the C118Y mutation. The presence of SRB and *Sw5*-non-infecting (SNI) TSWV was also observed some years ago in the same area on tomato and artichoke crops^36^. In those assays, *Sw-5b*-carrying tomatoes harbored only SRB strains, which had only the C118Y mutation. On the other hand, tomato varieties without the *Sw-5b *gene and artichokes were found infected by SNI or SRB strains, with the latter showing sometimes the T120N mutation^36^. Our observations, hence, further confirm that very likely no other mutations occurred in the SRB TSWV populations of southern Italy, such as C118F^35^, D122G^34^, or others^32,33^.

Our samples were grouped according to virus titer, i.e., ASY, SYM1, SYM2, and SYM3. We hypothesized that these groups could be considered representatives of different stages of TSWV infection, thus providing an intriguing scenario to inspect various levels of TSWV impact on the host transcriptome. PCA confirmed such a hypothesis as these groups clustered separately (Fig. 2A). Moreover, the number of differentially expressed genes (DEGs) increased as a function of the virus titer, reaching 44% of the whole transcriptome with the most severe infection (SYM3; Fig. 2B and C). Remarkably, correlation analysis unveiled that the expression of 45% of transcripts (over 14000) was significantly correlated to TSWV accumulation in leaf tissues. This phenomenon has been poorly documented in the literature. The increasing number of DEGs together with the progress of TSWV infection has been reported only in tomatoes carrying the *Sw-7 *resistance gene compared to a susceptible genotype^29^ and in the model plant *Arabidopsis thaliana*^37^. The verification of such a correlation by RT-qPCR with a different *Sw-5b*-tomato variety artificially inoculated with another SRB-TSWV isolate, allowed us to identify genes that can be considered good candidate markers of TSWV infection, such as *1-aminocyclopropane-1-carboxylic acid (ACC) synthase 2*, *Glutathione S-transferase*, *Abscisic acid stress ripening 5*, and *Late embryogenesis abundant (LEA) hydroxyproline-rich glycoprotein* (Fig. 5B).

In our experiment, TSWV infection had the most pronounced effects on photosynthesis and protein biosynthesis, which showed substantial down-regulation of many genes. Reasonably, these hampered pathways might be the major causes of growth stunting and yield losses of the crop. Also, in the secondary metabolism, carotenoid biosynthesis was deregulated (Figure S9), suggesting a possible negative impact on fruit quality. In line with our observation, a significant impact of TSWV on photosynthesis has been also reported in tomatoes lacking *Sw-5b*^24^ and other plant hosts such as peanuts and *A. thaliana *infected by SNI isolates^37,38^. Notably, scientific literature has shown that photosynthesis repression is a general effect in diverse compatible plant-virus interactions rather than a pathosystem-specific phenomenon. It has been reported that viral infections induce ultrastructural modifications of chloroplasts, down-regulation of chloroplast-related and photosynthesis-related genes, and interactions of viral proteins with nucleus-encoded chloroplast-targeted proteins^31,39,40^. In several cases, chloroplast-mediated defense responses against viruses have been described^40^. Up-regulation of photosynthesis-related genes has been observed in a TSWV-resistant tomato (*Sw-7 *gene) compared to a susceptible genotype^29^. Similarly, in a soybean variety carrying the *Rsv3 *resistance gene, chloroplast-related genes were induced in response to infection with soybean mosaic virus (SMV), especially in comparisons of an avirulent strain to a virulent one^41^.

While extensive literature has agreed on the detrimental effect of viral infections on photosynthesis, variable or contrasting effects have been observed on carbohydrate, hormone, and protein metabolisms^42^. In our research, down-regulation of genes of the plastidial, mitochondrial, and ribosomal machinery, peptide maturation, protein folding and translocation (especially in the chloroplast) suggested that protein biosynthesis was another pathway impaired by TSWV (Figure S5, S6, and S12). Information about the impact of viral infections on the ribosomes in plants is scanty. Early research has demonstrated a rapid decline in the concentration of chloroplast (70 S) and cytoplasmic (80 S) ribosomes in TSWV-infected tobacco leaves^43^. In tomatoes, citrus exocortis viroid (CEVd) has been found to provoke changes in the global polysome profiles, ribosome biogenesis, and a higher expression level of the ribosomal stress mediator *NAC082*^44^. On the other hand, for animal-infecting viruses, the interaction with ribosomes has been studied in more detail. Viruses need to manipulate ribosomes to accomplish their protein synthesis. They affect ribosome production and assembly as well as the recognition of mRNA by ribosomes, thus promoting viral protein synthesis and inhibiting the synthesis of host antiviral immune proteins^45^.

Our RNA-Seq analysis disclosed an enhanced expression of genes involved in pathogen-associated molecular pattern (PAMP)-triggered immunity (PTI) and effector-triggered immunity (ETI) (Figure S15). These networks are two interconnected branches of the plant’s innate immunity which can activate local as well as systemic defense responses, together with the post-transcriptional gene silencing machinery^46,47^. We also demonstrated that TSWV infection triggered a complex regulatory circuit including transcription factors (TFs) regulating the expression of defense genes. TF families such as WRKY and MYB showed the highest number of up-regulated genes (Figure S7). Both these TFs are strongly induced in plants upon viral infections, and they activate various signaling pathways, even with the participation of phytohormones^48,49^.

We did not observe substantial alterations of phytohormone genes, except for an overall down-regulation of some auxin-related genes, up-regulation of jasmonate-related genes (Figure S8), *phenylalanine ammonia lyase* (PAL) gene family, *p-coumarate: CoA ligase*, and *cinnamate-4-hydroxylase* (Figure S9).

## Conclusions

The tomato transcriptome was considerably rearranged upon TSWV infection (over 13000 differentially expressed genes, viz. up to 44% of the transcriptome), with deregulation of photosynthesis, protein biosynthesis, and induction of defense responses. Our observations on *Sw-5b*-carrying tomatoes infected by *Sw-5*-resistance-breaking TSWV overlapped with those on pathosystems lacking the *Sw-5b* gene and resistance-breaking mutation (e.g., C118Y) as well as with other plant-virus compatible interactions.

Moreover, this research demonstrated that the magnitude of transcriptional changes was proportional to the virus accumulation level (titer) in the leaf tissues as the expression of over 14000 genes (45% of the transcriptome) was significantly correlated to the virus titer.

## Methods

### Plant material collected for the RNA-Seq analysis

Leaf samples were collected from a field crop of tomato cv. Docet (carrying the *Sw-5b* resistance gene; Seminis, Bayer CropScience S.r.l., Milano, Italy) located at Stornara, Foggia, Apulia region, Italy (41°18’11.3” N 15°45’06.2” E). The field was surrounded by an olive grove, a vineyard, a peach grove, and a non-planted field. In the area, several tomato crops and other vegetables were cultivated. Sixty days after transplanting, young, fully expanded leaves were randomly sampled in the field (one leaf per plant). A total of 12 leaves with visible TSWV symptoms and six leaves from asymptomatic plants were collected. Symptomatic leaves showed typical TSWV symptoms such as ring spots and necrosis; plants bearing these leaves also showed stunting, ring spots on fruits, and necrosis on stems. Asymptomatic leaves did not show visible symptoms of TSWV, any other diseases or damage by pests. Leaflets were placed in 1.5 mL tubes, submerged in liquid nitrogen, and transferred to the laboratory where they were stored at −80 °C until use.

### RNA-Seq

Total RNA was extracted from the leaflets using Plant Total RNA Purification MiniKit (Fisher Molecular Biology, Trevose, PA, USA) according to the manufacturer’s instructions. Then, RNA was subjected to DNase digestion using TURBO DNA-free™ Kit (Thermo Fisher Scientific, Waltham, MA, USA), quantified by NanoDrop 1000 (Thermo Fisher Scientific), and its quality was checked by gel electrophoresis. RNA was sent to Macrogen Europe (Amsterdam, The Netherlands) for the library preparation and sequencing using TruSeq Stranded Total RNA with Ribo-Zero Plant Kit (Illumina). Samples were sequenced in HiSeq 2500 (Illumina) with a paired-end mode and 125 bp-read length, producing about 30 million reads per sample. Sequencing reads were submitted to NCBI Sequence Read Archive (SRA) under the BioProject ID PRJNA1134673 (https://www.ncbi.nlm.nih.gov/sra/PRJNA1134673).

Data analysis was conducted using the Galaxy platform version 20.01^50^ locally installed on a computer with a 16-core CPU and 64 GB RAM. Raw reads (FASTQ file format) were quality-checked using FASTQC v. 0.72 + galaxy1. Then, Trimmomatic v. 0.40^51^ was used to clean the reads: both 5’ and 3’ ends were trimmed if had an average Phred quality score lower than 20 in sliding windows of 4 nucleotides. Clean reads were mapped to the reference tomato genome (SL4.0; https://solgenomics.net) using Bowtie2 2.5.0 + galaxy0^52^ and counted using featureCounts 2.0.1 + galaxy2^53^. Differentially expressed genes (DEGs) were identified by DESeq2^54^ using a false discovery rate (FDR) threshold below 0.05. Gene ontology (GO) was retrieved from the Sol Genomics Network website and additional GO terms were obtained using Blast2GO 6.0.3, which was also used to perform an enrichment analysis of GO based on Fisher’s exact test (FDR < 0.05)^55^. Further gene annotations were obtained using BlastKOALA^56–59^ and MapMan 3.6.0RC1 with the mappings X4.4 (https://mapman.gabipd.org/mapman-download). Finally, the R package *fgsea *was used for the over-representation analysis^60^.

Pearson’s correlation procedure was used to correlate gene expression to TSWV titer. Plots were generated using R 4.3.0 (ISBN 3–900051-07-0; http://www.Rproject.org) and the *ggplot2 *3.4.0 package^61^ within RStudio 2023.03.1 build 446 (http://www.rstudio.com).

### Genome assembling of TSWV strain FGtomSRB

To determine the sequence of the TSWV strain infecting the tomato samples, which was designated ‘FGtomSRB’, sequencing reads were assembled into contigs using Velvet Galaxy version 1.2.10.3 with 21-mer hash length^62^. Contigs corresponding to the TSWV genome were identified by the blastn algorithm against the NCBI nr/nt database, limited to the virus taxon (taxid 10239). Then, open reading frames (ORFs) were identified by ORFinder (https://www.ncbi.nlm.nih.gov/orffinder) and the annotations were obtained by using blastn and blastx algorithms. The TSWV complete genome was deposited to NCBI GenBank under the accession numbers OR166266, OR166267, and OR166268 for the RNAs S, M, and L, respectively. The nucleotide polymorphism along the *Nsm *gene was checked to determine the resistance-breaking trait of the TSWV FGtomSRB strain, according to previous knowledge^32–35^.

The number of reads mappings to the TSWV genome was normalized (reads per million or RPM) and used to infer the virus accumulation (or virus titer) in the leaf tissues. Based on the virus titer, the 18 samples were assembled into four groups (see below), which were used to inspect differential gene expression.

The presence of any other virus in the leaf samples was ascertained using a bioinformatic pipeline based on contig assembling and blast search against NCBI database^63^.

### Greenhouse experiment

A greenhouse experiment (natural lighting, 27/19 ± 3 °C day/night) was carried out to validate the gene expression × virus titer correlation observed by RNA-Seq. For a more robust validation, the experiment was conducted with a different *Sw-5b*-carrying tomato variety, Impact F_1_ (ISI Sementi, Fidenza, Parma, Italy), and another SRB-TSWV isolate, T-1012 (obtained from the Institute’s PlaVit collection). Twenty-day-old tomato seedlings were transplanted into 1.4 L-plastic pots (one per pot) containing rodhic, chromic, calcic luvisol soil (‘terra rossa’ soil with about 1% organic carbon). Five groups (A to E) of six plants each were rub-inoculated on the second true leaf with TSWV isolate T-1012. Each plant group was inoculated on a different day to obtain different virus accumulation (titer) levels at the sampling time, which was done in one single day from all the plant groups. Group A was not inoculated, while groups B to E were inoculated on April 28^th^, May 2^nd^, 4th, and 5^th^ (2024), respectively, and the sampling (one leaflet per plant from the systemically infected leaf) was done on May 10^th^. Plants were irrigated as needed and no phytosanitary treatments were made.

The progress of TSWV symptoms over time was visually evaluated according to a 0–4 empirical scale designed in this study, where 0 = no symptoms, 1 = yellowing, 2 = growth reduction and leaf distortion, 3 = systemic development of necrotic lesions on stems and leaves, and 4 = dead plant.

### RT-qPCR

Leaflets from the greenhouse experiment were subjected to RNA extraction immediately after sampling. Total RNA was extracted from approximately 100 mg of leaf tissues as described above. Then, 1 µg of total RNA was reverse transcribed with the Maxima H Minus First Strand cDNA Synthesis Kit (including dsDNase; Thermo Fisher Scientific) according to the manufacturer’s instructions. RT-qPCR reactions were carried out with 10 ng of cDNA, 400 nM of each forward and reverse primers and PowerUp™ SYBR™ Green Master Mix (Thermo Fisher Scientific) assembled in a total volume of 20 µL. The reactions were performed in a StepOnePlus™ Real-Time PCR System (Thermo Fisher Scientific) using the fast-cycling profile recommended by the manufacturer. The annealing temperature was 60 °C for all the primer pairs. Data were expressed as gene expression relative to the housekeeping gene (*actin*) using the 2^−ΔΔ^CT method^64^ and the correction for the PCR efficiency. This calculation was made by the StepOne™ Software v.2.3 (Thermo Fisher Scientific). The housekeeping gene was chosen according to Mascia, et al.^65^ and an in-house check of the stability using the RNA-Seq data of this work.

Fifteen genes were randomly selected among those with the highest positive or negative correlation rate (R^2^) between the expression level and the virus titer in the RNA-Seq experiment. Virus titer, or accumulation of TSWV in the tissues, was also measured. Primers for plant and viral genes employed in the RT-qPCR assay are listed in Table S1. They were retrieved from the literature or designed for this study using Primer3^66^ or Primer-BLAST tools (https://www.ncbi.nlm.nih.gov/tools/primer-blast/).

## Electronic supplementary material

Below is the link to the electronic supplementary material.

Supplementary Material 1

## Data Availability

RNA-Seq data generated during the current study are available at NCBI Sequence Read Archive (SRA) under the BioProject ID PRJNA1134673 (https://www.ncbi.nlm.nih.gov/sra/PRJNA1134673). The complete genome of tomato spotted wilt virus strain FGtomSRB determined during the current study is available at NCBI GenBank under the accession numbers OR166266, OR166267, and OR166268 for the RNAs S, M, and L, respectively.
