# Supplementary material for: Resistance-breaking strains of tomato spotted wilt virus hamper photosynthesis and protein synthesis pathways in a virus accumulation-dependent manner in Sw5-carrying tomatoes
Source: Sci Rep. 2025 Jan 29;15:3630. doi: 10.1038/s41598-025-88028-x (PMC11779902; doi:10.1038/s41598-025-88028-x)
Supplement: Supplementary file 1 — Supplementary Material 1 [file 41598_2025_88028_MOESM1_ESM.pdf]

SUPPLEMENTAL MATERIAL OF

Resistance-breaking strains of tomato spotted wilt virus hamper photosynthesis and protein synthesis pathways in a virus accumulation-dependent manner in *Sw5*-carrying tomatoes

Maria Isabella Prigigallo, Ugo Picciotti, Giovanni Bubici*

Istituto per la Protezione Sostenibile delle Piante, Consiglio Nazionale delle Ricerche, via Amendola 165/A, 70126 Bari, Italy.

* Corresponding author: Giovanni Bubici, giovanninicola.bubici@cnr.it

Table S1. Primers used in the reverse-transcription quantitative PCR assay of the greenhouse experiment.

Gene		Sequence (5'->3')	Amplicon length (bp)
**Solyc01g095080.3.1**	1-aminocyclopropane-1-carboxylic acid (ACC) synthase 2	F. ATTGGGCACCACTTTGGACA<br>R: TGGCTGCGTAGATTTCGTCA	98
**Solyc10g054010.1.1**	BZIP transcription factor	F: GGCTGAAAACTCAATCCTAAGAGC<br>R: TGTTGCCGTTGTAGCTCAAGA	98
**Solyc04g071600.3.1**	Abscisic acid stress ripening 5	F: ACATCACCGTTTGTTCCACC<br>R: TGGAGATGGCTATGGTGCTT	90
**Solyc06g062460.3.1**	bHLH transcription factor 136	F: CCAGCAAGCAAGTTCTTCGG<br>R: AGAGCTCACTGGCCTGAATG	109
**Solyc04g072280.3.1**	Laccase	F: CATCACAATTTCCCCGGGTG<br>R: ACGCTATTGTAGGCTTTCGC	96
**Solyc10g081980.2.1**	Late embryogenesis abundant (LEA) hydroxyproline-rich glycoprotein	F: ATCGACATGGACGGGGAAG<br>R: GAGGACGATGAGGATGGTGA	101
**Solyc10g079170.2.1**	Leucine-rich repeat receptor-like (LRR-RL) tyrosine-protein kinase	F: ACAAAGGGCTCTGTGGTGAG<br>R: ACCAGAACCAACAACAGCCA	113
**Solyc07g053480.4.1**	Ras-related protein	F: ATACCGTGTCGTGAGCAAGA<br>R: TGCTGAAATGTCGTCCTTGC	103
**Solyc08g083320.4.1**	Starch synthase	F: ACGTGGGGTTGATCGTGTTT<br>R: AATCCAGTCCAGCTTTGGGG	103
**Solyc05g010470.4.1**	WD40 repeat	F: GGGTTCTCGCCTGATTGTTC<br>R: ACATCCCCAAGTTCCATGCT	107
**Solyc07g056280.3.1**	WRKY transcription factor 30	F: ACATGGAGGGTCAATGGATCA<br>R: TGTGGACAAACAACTTCAGAGG	90
**Solyc07g056420.4.1**	Glutathione S-transferase	F: CCCCTTTGCTCCCTTCTGAT<br>R: GTTCGTCTCCCGTTGCTGTA	90
**Solyc00g500066.1.1**	Photosystem I assembly protein Ycf4	F: TTTCGTTGGGGATTTCCGGG<br>R: ACACGACGAGCATAAATACCCT	110
**Solyc00g500322.1.1**	ATP synthase subunit b	F: TTCGAAATTCAGAAGAACTGCGT<br>R: AAACTGCTCGGCTTCGGTTT	95
**Solyc02g080890.3.1**	WRKY transcription factor 6	F: CAGTCCGAAAACAGGTGCAA<br>R: CATGGCTGCTGGTGGTAATG	98
**Solyc03g078400**	Actin (housekeeping)	F: AGGCAGGATTTGCTGGTGATGATGCT<br>R: ATACGCATCCTTCTGTCCCATTCCGA	107
	Tomato spotted wilt virus, non-structural protein (NSm gene) (Prigigallo et al. 2019)	F: TTTGGAACCTATGAATCTGATTC<br>R: CCGTTGCCAACAAAAAGATCATT	122

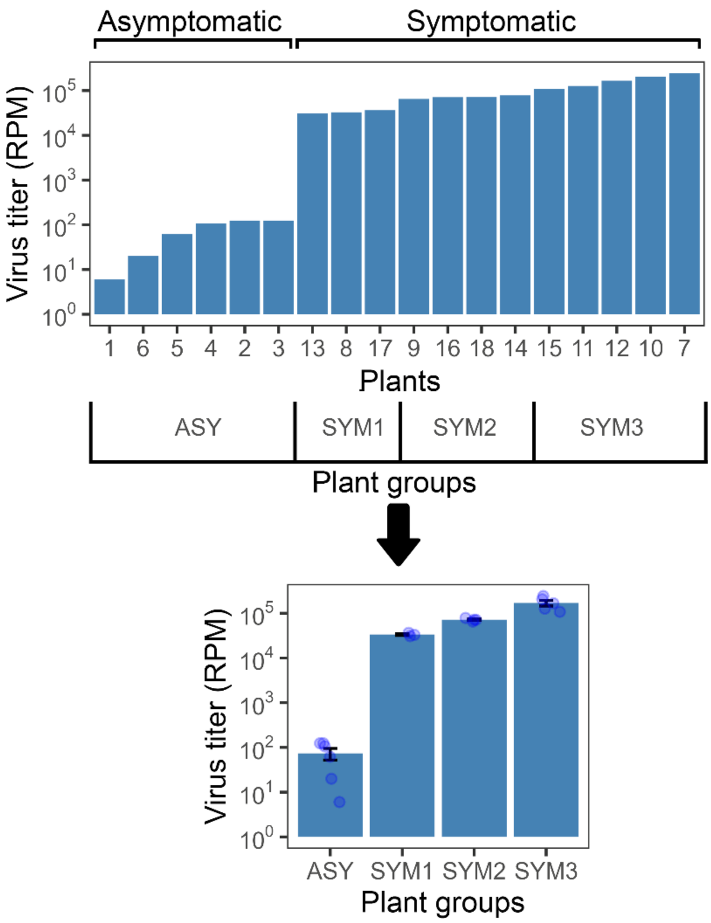

Figure S1. Accumulation of tomato spotted wilt virus (TSWV) in leaves of tomato plants sampled from the field. Plants were grouped according to their virus titer. Virus titer is expressed as reads per million (RPM) mapping to the TSWV genome.

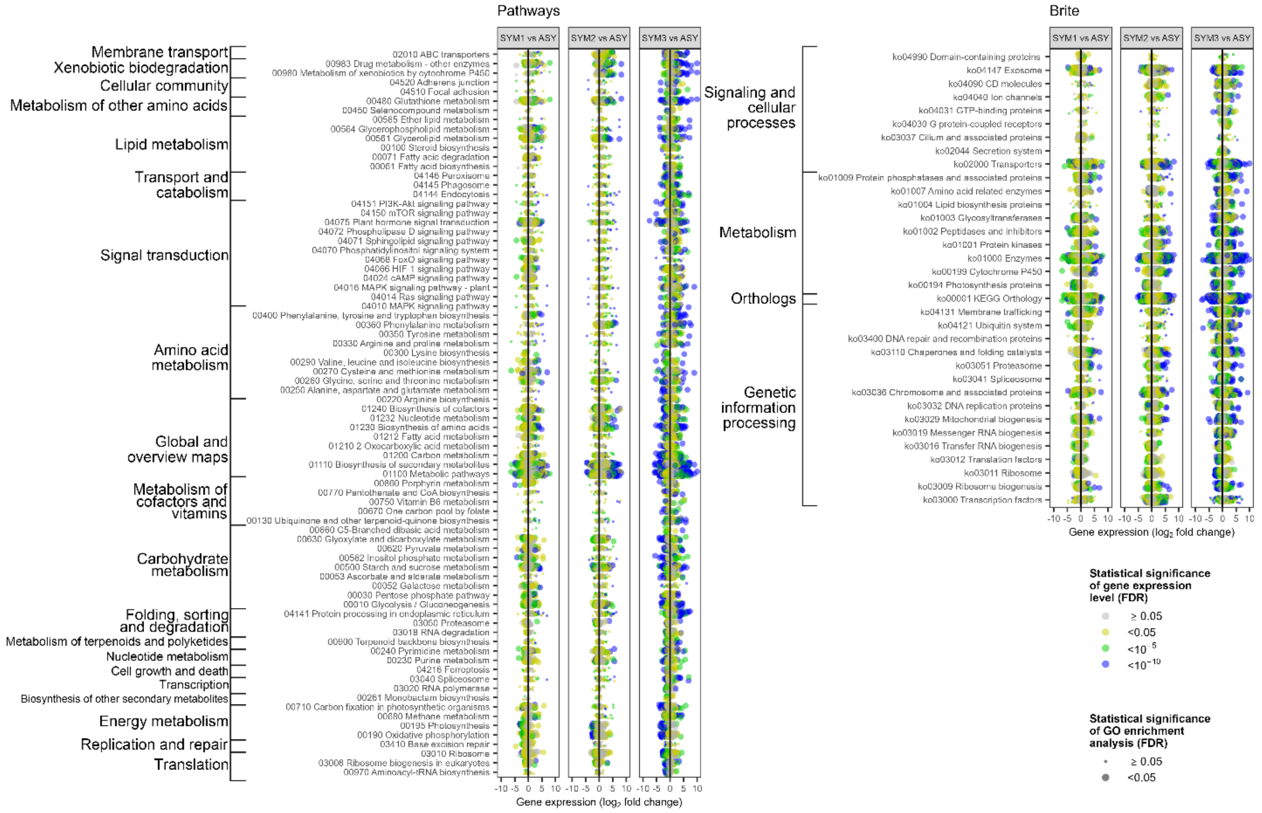

Figure S2. KEGG annotation of genes differentially expressed in tomato plants as revealed by RNA-Seq. Plant groups ASY, SYM1, SYM2, and SYM3 differed for the titer of tomato spotted wilt virus as depicted in Figure 1.

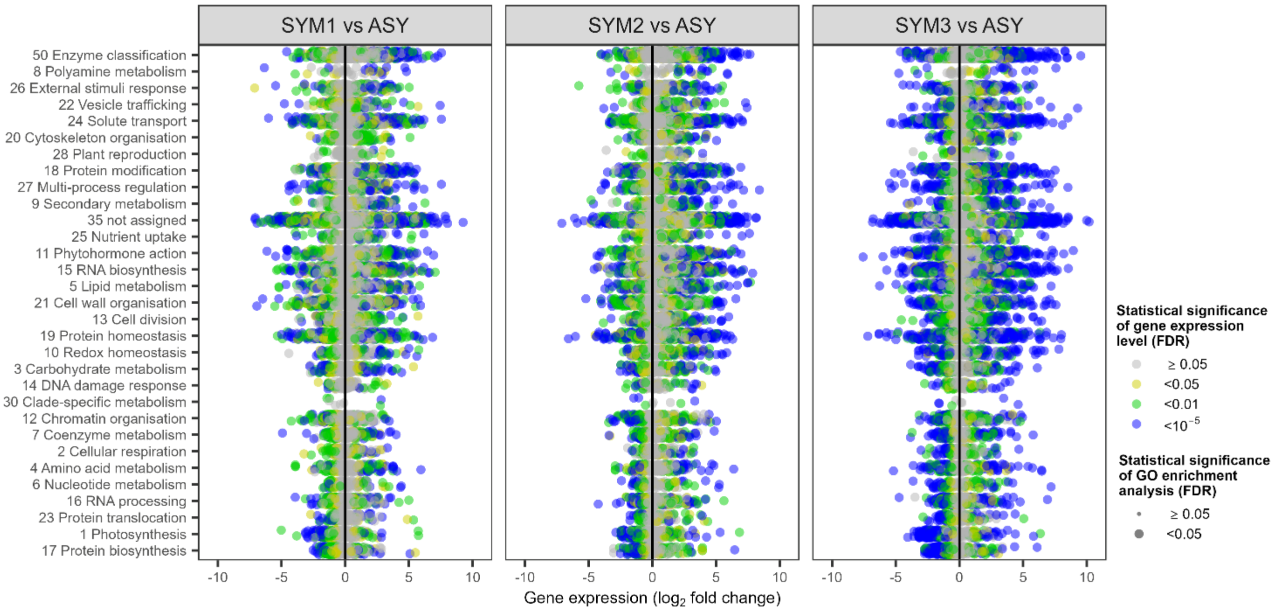

Figure S3. MapMan annotation of genes differentially expressed in tomato plants as revealed by RNA-Seq. Plant groups ASY, SYM1, SYM2, and SYM3 differed for the titer of tomato spotted wilt virus as depicted in Figure 1.

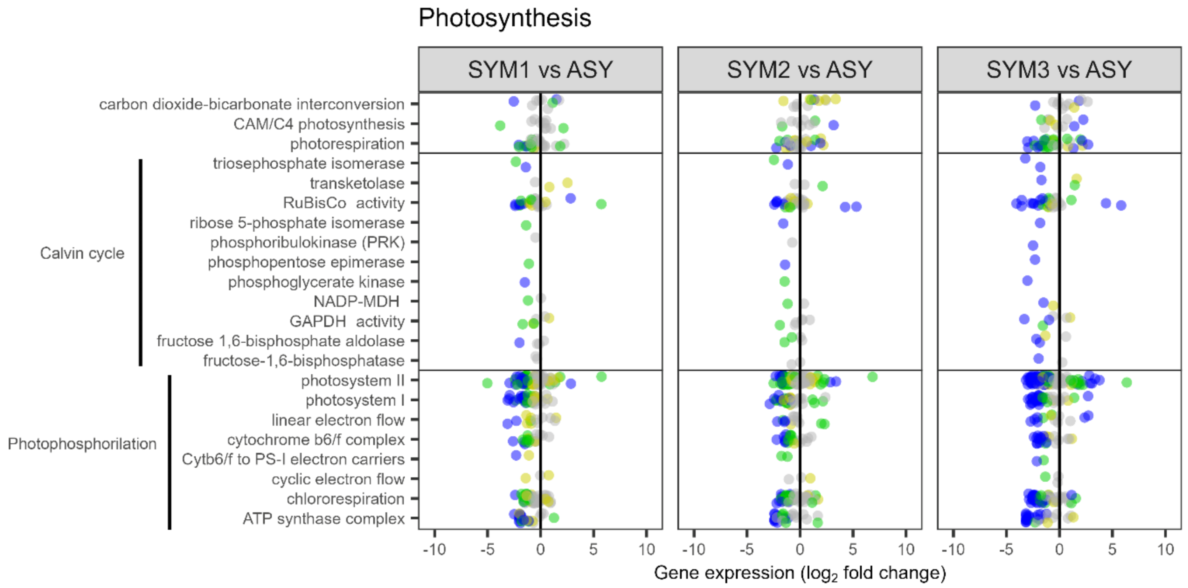

Figure S4. Photosynthesis pathway annotation (MapMan) of genes differentially expressed in tomato plants as revealed by RNA-Seq. Plant groups ASY, SYM1, SYM2, and SYM3 differed for the titer of tomato spotted wilt virus as depicted in Figure 1.

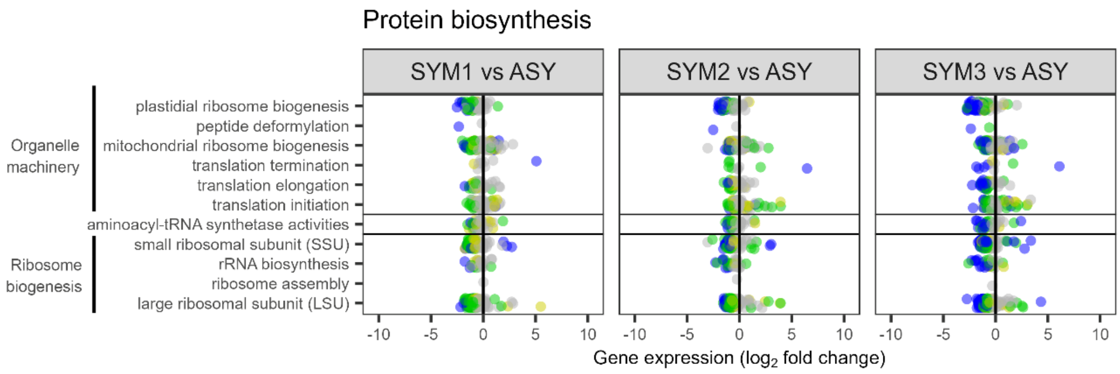

Figure S5. Protein biosynthesis pathway annotation (MapMan) of genes differentially expressed in tomato plants as revealed by RNA-Seq. Plant groups ASY, SYM1, SYM2, and SYM3 differed for the titer of tomato spotted wilt virus as depicted in Figure 1.

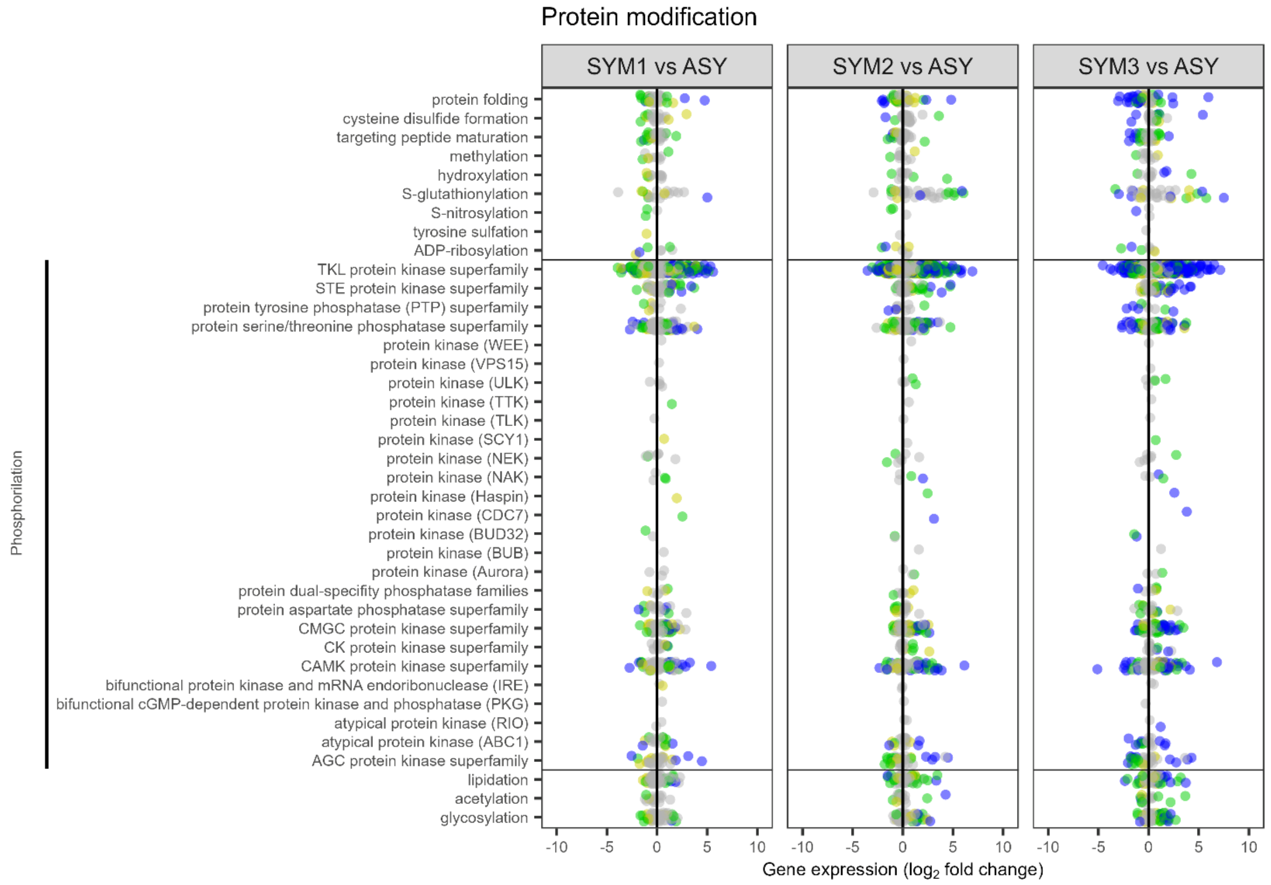

Figure S6. Protein modification pathway annotation (MapMan) of genes differentially expressed in tomato plants as revealed by RNA-Seq. Plant groups ASY, SYM1, SYM2, and SYM3 differed for the titer of tomato spotted wilt virus as depicted in Figure 1.

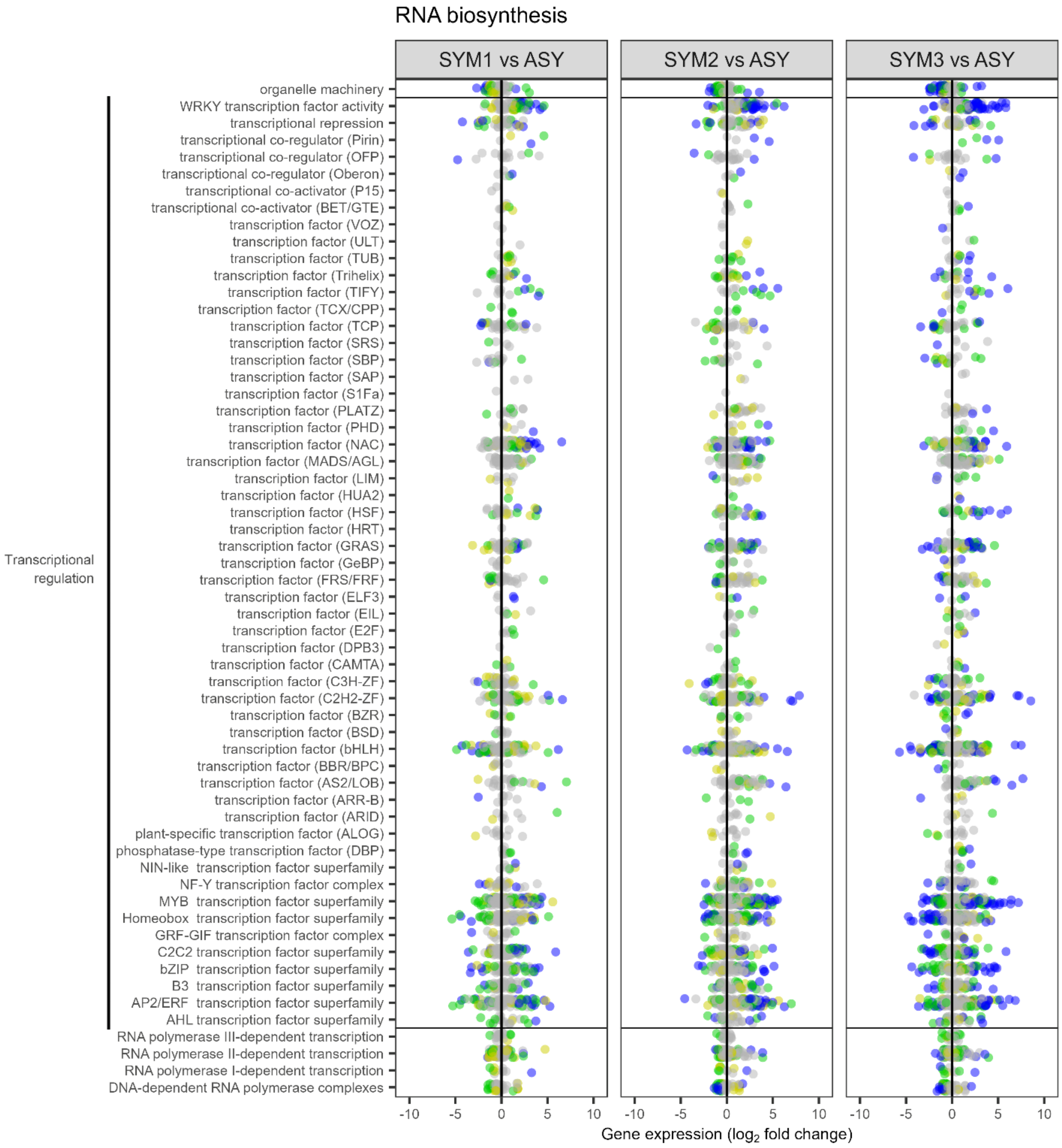

Figure S7. RNA biosynthesis pathway annotation (MapMan) of genes differentially expressed in tomato plants as revealed by RNA-Seq. Plant groups ASY, SYM1, SYM2, and SYM3 differed for the titer of tomato spotted wilt virus as depicted in Figure 1.

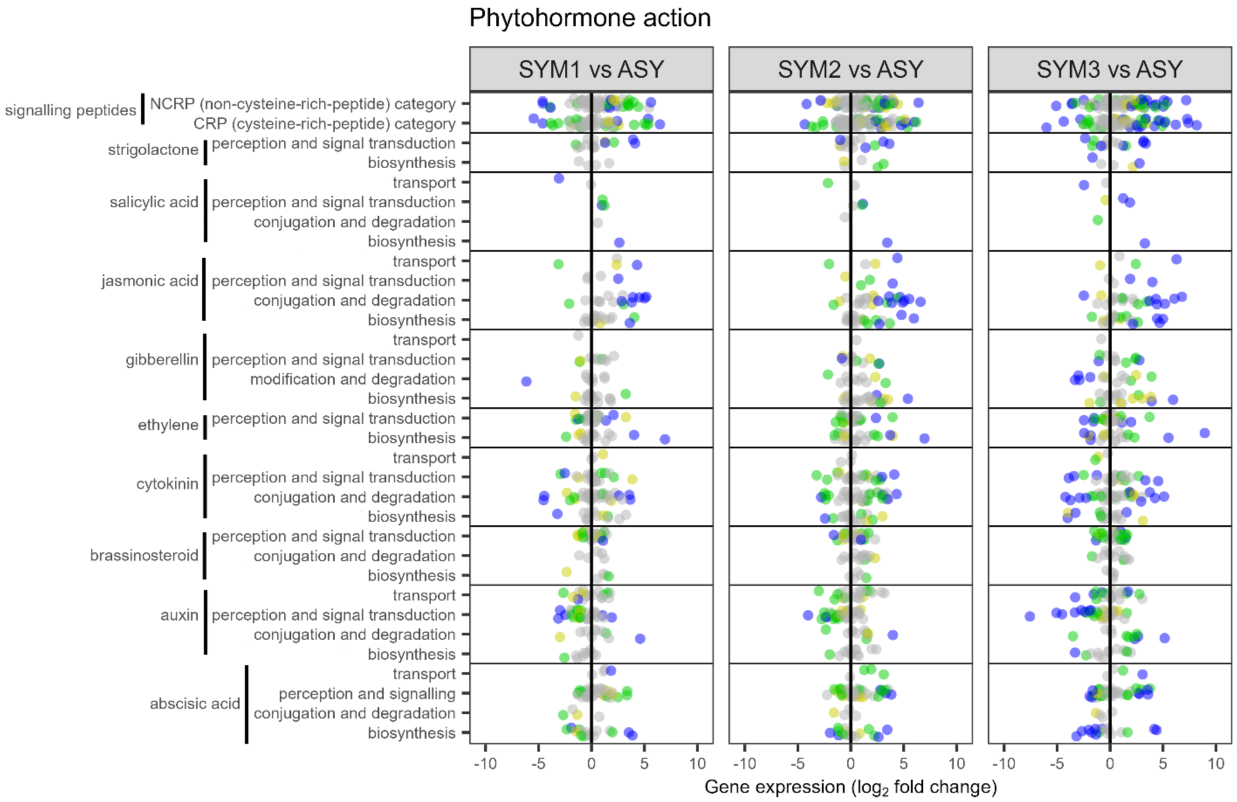

Figure S8. Phytohormone pathway annotation (MapMan) of genes differentially expressed in tomato plants as revealed by RNA-Seq. Plant groups ASY, SYM1, SYM2, and SYM3 differed for the titer of tomato spotted wilt virus as depicted in Figure 1.

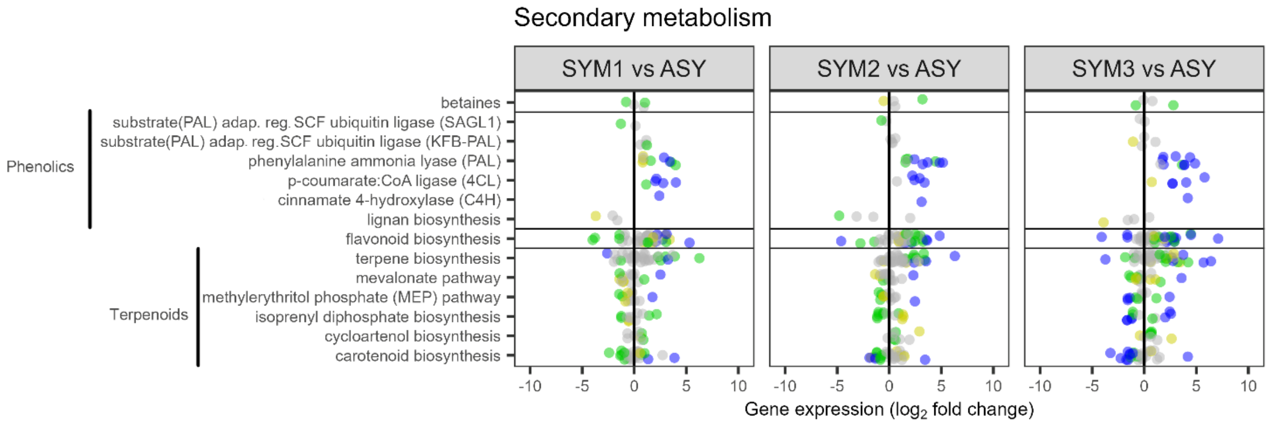

Figure S9. Secondary metabolism annotation (MapMan) of genes differentially expressed in tomato plants as revealed by RNA-Seq. Plant groups ASY, SYM1, SYM2, and SYM3 differed for the titer of tomato spotted wilt virus as depicted in Figure 1.

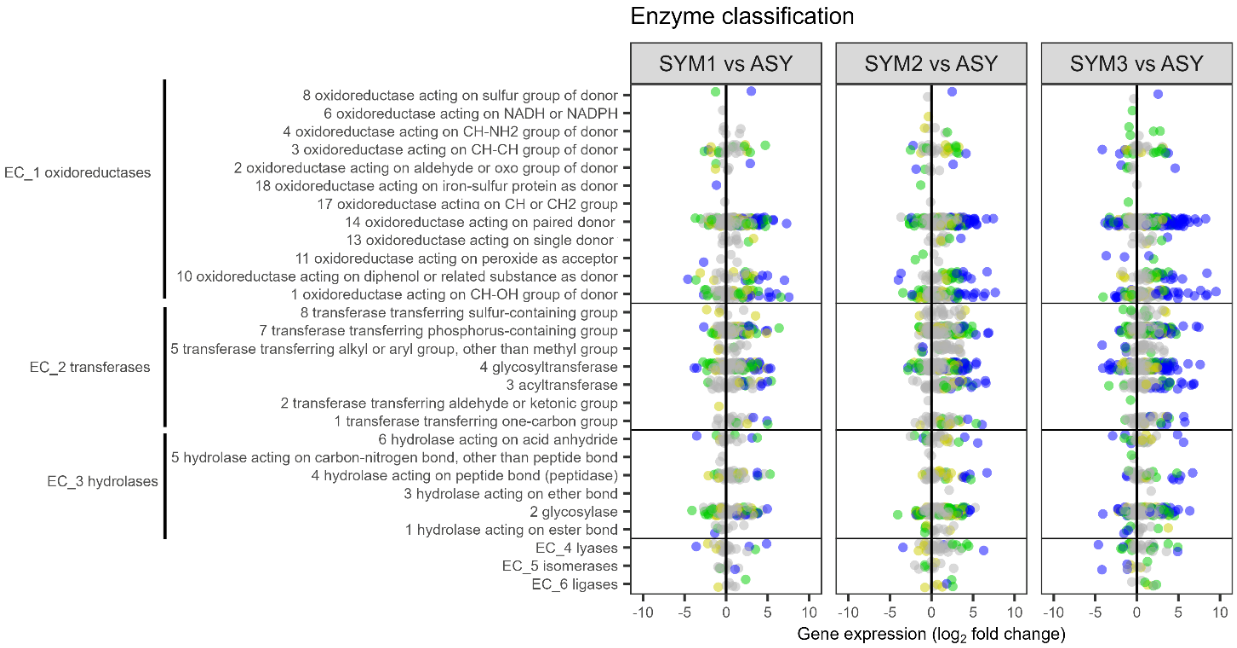

Figure S10. Enzyme annotation (MapMan) of genes differentially expressed in tomato plants as revealed by RNA-Seq. Plant groups ASY, SYM1, SYM2, and SYM3 differed for the titer of tomato spotted wilt virus as depicted in Figure 1.

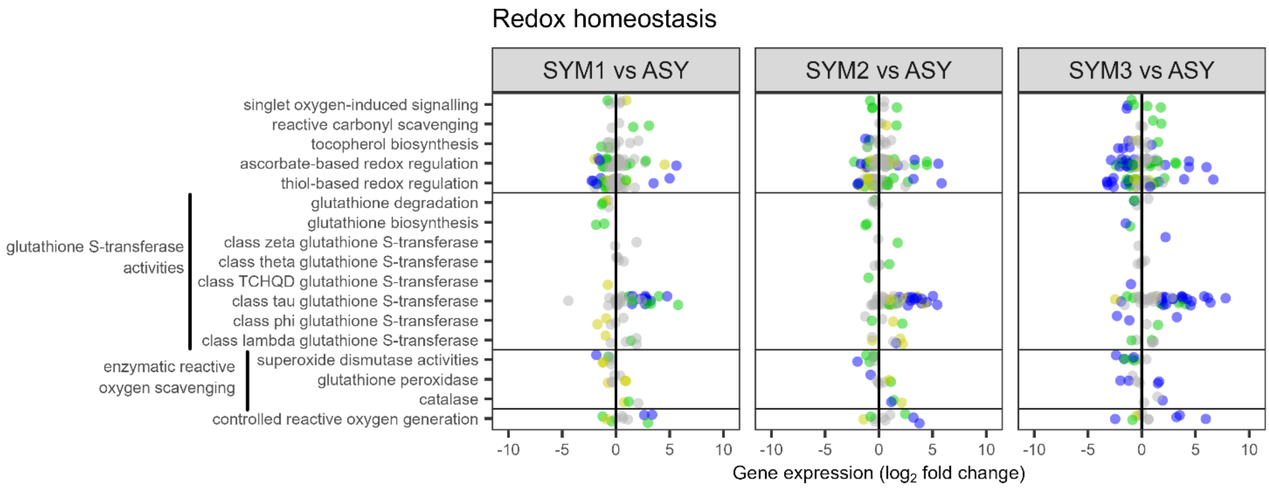

Figure S11. Redox homeostasis pathway annotation (MapMan) of genes differentially expressed in tomato plants as revealed by RNA-Seq. Plant groups ASY, SYM1, SYM2, and SYM3 differed for the titer of tomato spotted wilt virus as depicted in Figure 1.

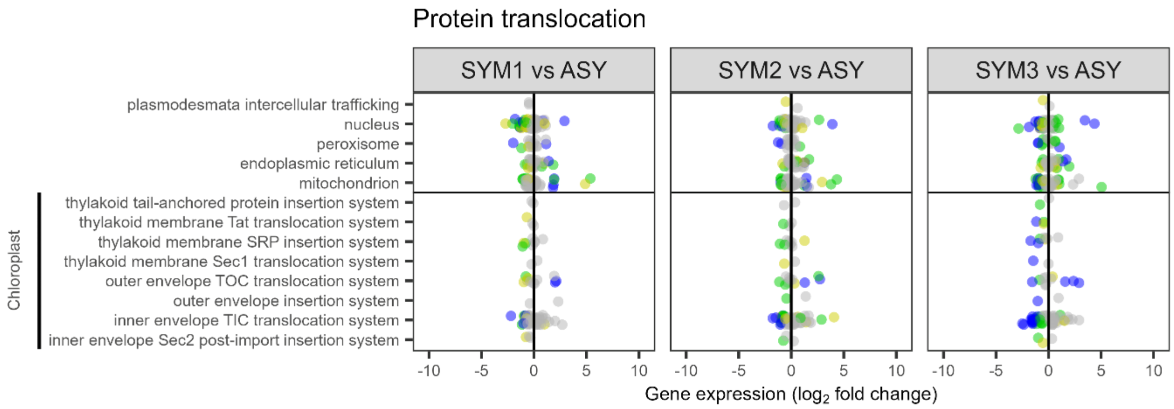

Figure S12. Protein translocation pathway annotation (MapMan) of genes differentially expressed in tomato plants as revealed by RNA-Seq. Plant groups ASY, SYM1, SYM2, and SYM3 differed for the titer of tomato spotted wilt virus as depicted in Figure 1.

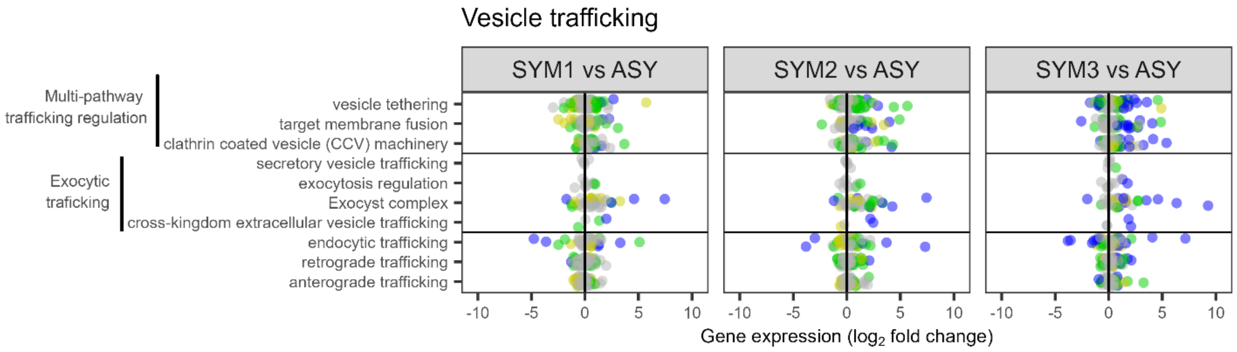

Figure S13. Vesicle trafficking pathway annotation (MapMan) of genes differentially expressed in tomato plants as revealed by RNA-Seq. Plant groups ASY, SYM1, SYM2, and SYM3 differed for the titer of tomato spotted wilt virus as depicted in Figure 1.

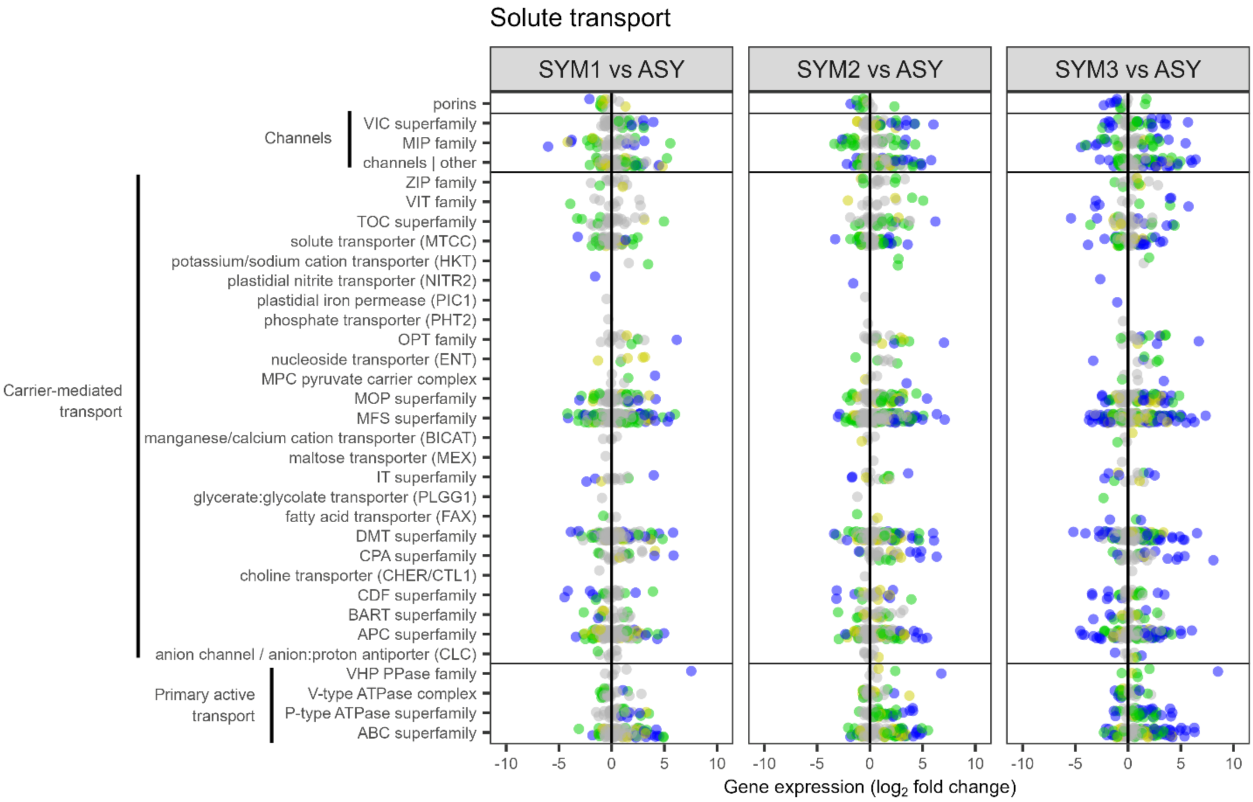

Figure S14. Solute transport pathway annotation (MapMan) of genes differentially expressed in tomato plants as revealed by RNA-Seq. Plant groups ASY, SYM1, SYM2, and SYM3 differed for the titer of tomato spotted wilt virus as depicted in Figure 1.

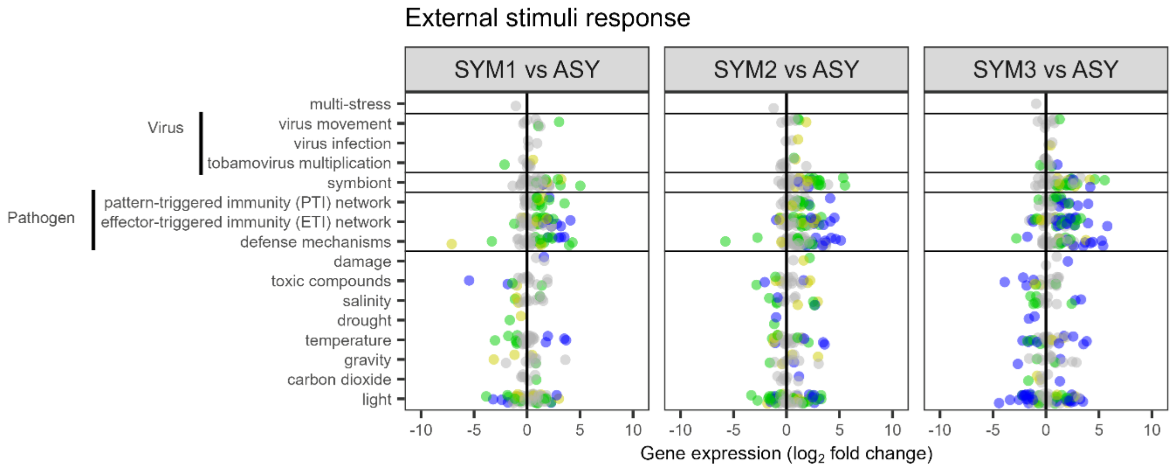

Figure S15. External stimuli response pathway annotation (MapMan) of genes differentially expressed in tomato plants as revealed by RNA-Seq. Plant groups ASY, SYM1, SYM2, and SYM3 differed for the titer of tomato spotted wilt virus as depicted in Figure 1.

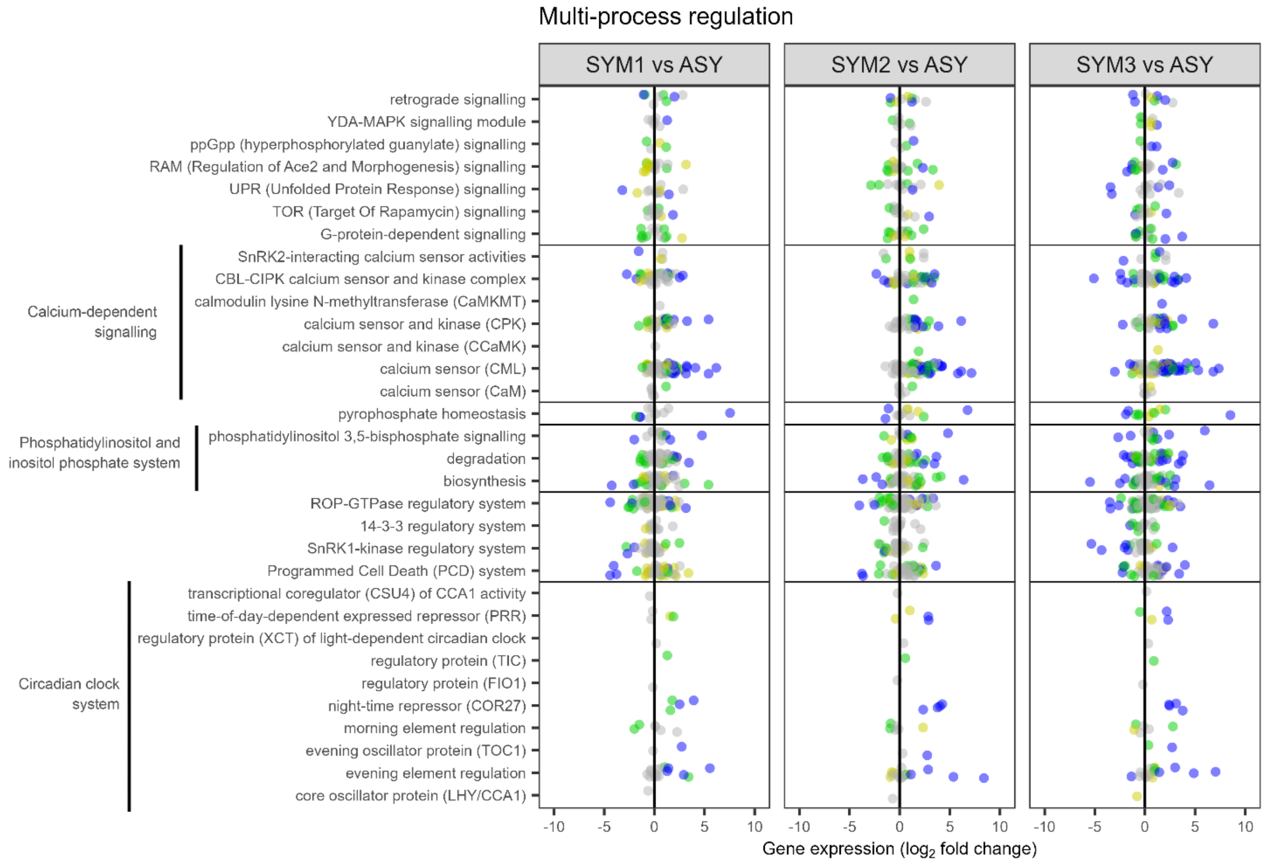

Figure S16. Multi-process regulation pathway annotation (MapMan) of genes differentially expressed in tomato plants as revealed by RNA-Seq. Plant groups ASY, SYM1, SYM2, and SYM3 differed for the titer of tomato spotted wilt virus as depicted in Figure 1.

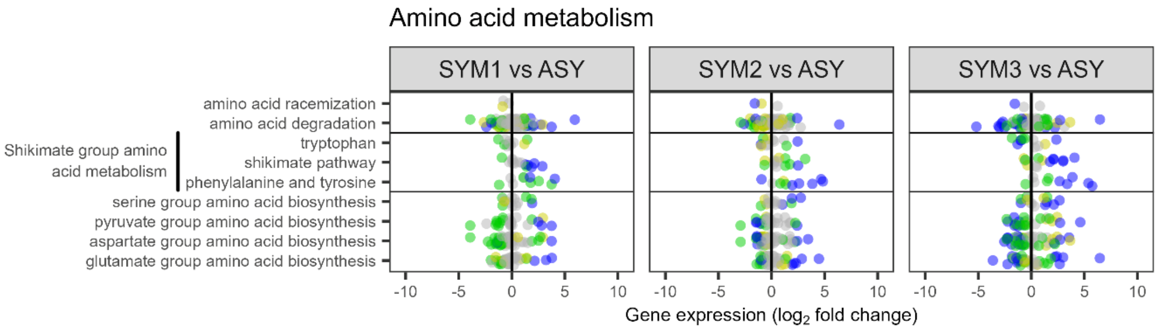

Figure S17. Amino acid metabolism annotation (MapMan) of genes differentially expressed in tomato plants as revealed by RNA-Seq. Plant groups ASY, SYM1, SYM2, and SYM3 differed for the titer of tomato spotted wilt virus as depicted in Figure 1.

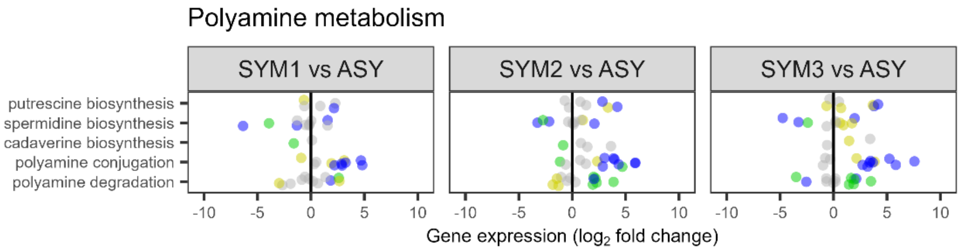

Figure S18. Polyamine metabolism annotation (MapMan) of genes differentially expressed in tomato plants as revealed by RNA-Seq. Plant groups ASY, SYM1, SYM2, and SYM3 differed for the titer of tomato spotted wilt virus as depicted in Figure 1.

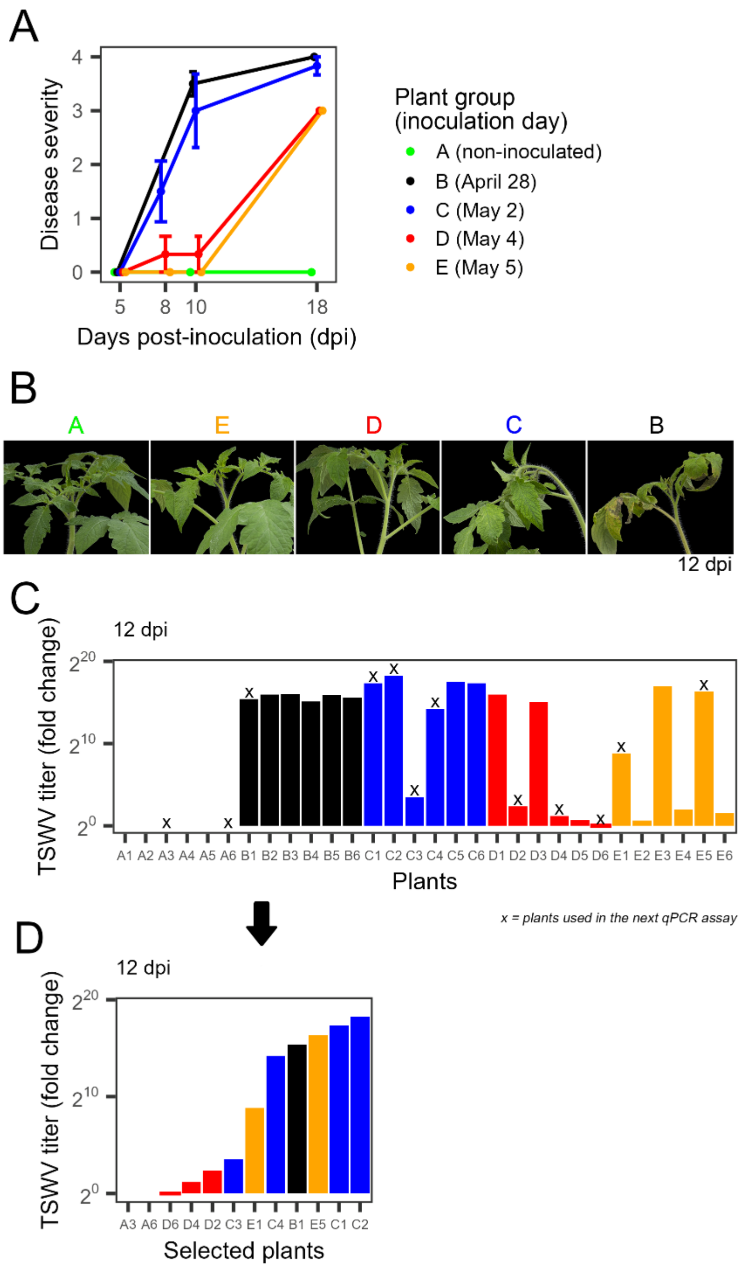

Figure S19. Greenhouse experiment: progress over time of tomato spotted wilt virus (TSWV) symptom severity (A) in different plant groups artificially inoculated at different time points; symptoms of TSWV at two dates in different plant groups (B); virus titer measured by qPCR on May 10 in all the plants (C) and only those selected to have a wide virus titer range for the following qPCR assay (D).
